# Peroxisome-associated *Sgroppino* links fat metabolism with survival after RNA virus infection in *Drosophila*

**DOI:** 10.1038/s41598-019-38559-x

**Published:** 2019-02-14

**Authors:** Sarah H. Merkling, Human Riahi, Gijs J. Overheul, Annette Schenck, Ronald P. van Rij

**Affiliations:** 1 Department of Medical Microbiology, Radboud Institute for Molecular Life Sciences, Radboud University Medical Center, Nijmegen, The Netherlands; 2 Department of Human Genetics, Donders Institute for Brain, Cognition and Behaviour, Radboud University Medical Center, Nijmegen, The Netherlands; 3 Present Address: Insect-Virus Interactions Group, Department of Genomes and Genetics, Institut Pasteur, Paris, France

## Abstract

The fruit fly *Drosophila melanogaster* is a valuable model organism for the discovery and characterization of innate immune pathways, but host responses to virus infection remain incompletely understood. Here, we describe a novel player in host defense, *Sgroppino* (*Sgp*). Genetic depletion of *Sgroppino* causes hypersensitivity of adult flies to infections with the RNA viruses Drosophila C virus, cricket paralysis virus, and Flock House virus. Canonical antiviral immune pathways are functional in *Sgroppino* mutants, suggesting that *Sgroppino* exerts its activity via an as yet uncharacterized process. We demonstrate that *Sgroppino* localizes to peroxisomes, organelles involved in lipid metabolism. In accordance, *Sgroppino*-deficient flies show a defect in lipid metabolism, reflected by higher triglyceride levels, higher body mass, and thicker abdominal fat tissue. In addition, knock-down of *Pex3*, an essential peroxisome biogenesis factor, increases sensitivity to virus infection. Together, our results establish a genetic link between the peroxisomal protein Sgroppino, fat metabolism, and resistance to virus infection.

## Introduction

Viruses are the most abundant biological entities on earth, capable of infecting all cellular life forms^1^. Being intracellular parasites, viruses exploit host cellular machineries and pathways at every step of their replication cycle. As a consequence, a myriad of host defense mechanisms have evolved that directly or indirectly restrict viral replication.

Cellular organelles are exploited by viruses for entry, replication, and assembly^2^. For example, many viruses enter the cell through endosomal compartments^3^, several DNA and RNA viruses remodel the ER network or other intracellular membranes into replication organelles for genome replication^4–6^, and enveloped viruses use cellular membranes for their assembly^2^. Yet, cellular organelles also play important roles at the other side of the virus-host interface, as they are important sites for immune signaling in mammals^7^. For instance, Toll-like receptors at the plasma membrane and in endosomal compartments patrol the extra-cellular environment to detect pathogen-associated molecular patterns (PAMPs)^8^, whereas mitochondrial membranes are sites for immune signaling via MAVS (mitochondrial antiviral signaling protein) after sensing of viral RNA by RIG-I-like receptors in the cytoplasm^8^.

The fruit fly *Drosophila melanogaster* is a powerful model organism for the identification and characterization of host defense mechanisms^9–11^, by virtue of its well-annotated genome and vast genetic toolbox. RNA interference (RNAi), for example, is a major antiviral mechanism that is initiated by processing of viral double-stranded RNA into small interfering RNAs by Dicer-2. These small RNAs guide cleavage of viral RNA by the Argonaute-2 containing RNA induced silencing complex^12,13^. In addition, virus infection activates signaling pathways to induce transcriptional responses, such as the Jak-Sat pathway and the NF-κB-dependent Toll and Imd pathways^14–21^. These pathways seem to participate in antiviral defense in a virus-specific manner and their relative importance may also depend on the route of inoculation. For instance, Toll signaling is required for resistance to oral, but not systemic infection^20^. Finally, essential cellular processes, such as autophagy and the heat shock response, are also required for resistance to virus infection^22–25^.

Here, we describe a novel player in host defense against RNA viruses in *Drosophila*, which we named *Sgroppino*. *Sgroppino*-deficient flies are hypersensitive to RNA virus infection, which in the case of Drosophila C virus was associate with higher levels of replication. We demonstrate that *Sgroppino* localizes to peroxisomes and that knock-down of the peroxisome biogenesis factor *Pex3* causes hypersensitivity to virus infection. In agreement with its predicted function, *Sgroppino* mutant flies exhibit defects in lipid metabolism. Altogether, our data demonstrate that *Sgroppino* participates in host response, possibly by affecting lipid metabolism in peroxisomes.

## Materials and Methods

### Fly strains and husbandry

Flies were raised on standard cornmeal-agar medium at 25 °C in a light/dark cycle of 12 h/12 h. All experiments, including RNAi mediated knock-downs were performed at 25 °C. The *CG13091/Sgroppino* mutant (referred to as *Sgp*^−/−^) contains a P{EPgy2} transposon insertion in the 3′-untranslated region of the CG13091 transcript^26^ (Supplementary Fig. 1B). *Sgp*^−/−^ and Arm-Gal4 driver lines were obtained from Bloomington Stock Center (stock numbers 15973 and 1561). Flies expressing *Sgp*^RNAi^ and *Ago2*^RNAi^ hairpins under control of UAS were obtained from the Vienna Drosophila Stock center (stock no. 100943 and 49473) and UAS-*Pex3*^RNAi^ and UAS-*GFP*^RNAi^ flies from the NIG-Fly Stock Center (stock no. 6859R-4 and GFP-IR-1). *Hsf*^4^ and *CnBw* fly lines have been described previously^25^. We used *y*^1^*w*^1^ flies as control for *Sgp*^−/−^ in all experiments.

*In vivo* RNAi experiments were performed by crossing GMR-Gal4, UAS-*Diap1*^RNAi^/CyO; *Ago2*^321^/TM6, Sb virgins^27^ with male UAS-*Sgp*^RNAi^ flies, UAS-*Ago2*^RNAi^ flies, or control flies containing the attP landing site that was used to introduce the RNAi-inducing transgenes *(y*^1^*v*^1^*; attP2*; Bloomington stock no. 36303). The eye phenotype was assessed in three to five-day-old female F1 offspring containing the TM6, Sb balancer and lacking the CyO balancer.

The 6 single nucleotide polymorphisms (SNPs) sites in the *pastrel* locus of *Sgroppino* mutants and *y*^1^*w*^1^ flies were determined by sequencing, as previously described^28^. *Sgp* mutants contained the following SNPs (genome positions: 3L:7,350,452 G, 3L:7,350,453 G, 3L:7,350,895 T, 3L:7,352,880 C) and two SNPs in introns (3L:7,351,494 T, 3L:7,352,966 G). With the exception of the SNP at position 3L:7,352,280 T, the *y*^1^*w*^1^ control flies contained identical SNPs as *Sgp* mutants, including the SNP at the 3 L:7,350,895 position that is strongly associated with resistance to DCV infection^29^.

### Starvation and heat shock assay

For the starvation assay, three to five-day-old flies were transferred, without using CO_2_ anesthesia, from standard fly food to starvation medium, consisting of distilled water jellified with 0.66% agar (wt/vol) (adapted from^30^). For the heat shock assay, three to five-day-old flies were incubated at 35 °C for 4 days. Flies were transferred to fresh medium every 2 days, and survival was assessed daily.

### Weight measurement

Embryos were collected on apple juice-agar plates as previously described^28^. Fifty embryos were transferred to a single culture vial containing standard cornmeal-agar medium and cultured at 25 °C. Five to seven-day-old flies were collected and frozen in groups of ten individuals of a single sex. The weight of each group was determined on a precision scale and expressed as mass per individual fly.

### Time to pupation assay

Fifty embryos were grown on standard cornmeal-agar medium at 25 °C, as described for weight measurement. The appearance of pupae was scored twice a day.

### Quantification of triglycerides

Three pools of two flies were homogenized in 150 μL lysis buffer (1% NP-40 in PBS). Samples were heated for 5 minutes at 90 °C and allowed to cool down at room temperature; this step was repeated twice. Debris was pelleted by centrifugation at 16,000 g for 2 min, and supernatant was transferred to a new tube. Total protein concentration was quantified using the Pierce BCA protein assay kit (Thermo Scientific) on 25 μL of undiluted lysate, following the manufacturer’s instructions. Triglycerides were measured with the Triglyceride Quantification kit (BioVision), following the manufacturer’s instructions using a 1:40 dilution of the same lysate. Colorimetric measurements were performed at 570 nm using a Biotek Synergy 2 plate reader. All measurements were performed in triplicate, and triglyceride levels were normalized against protein levels.

### Quantification of lipid peroxidation

Peroxidized lipids were quantified using the lipid peroxidation kit (K739, BioVision) following the manufacturer’s instructions. Three pools of 20–40 young (2–4 days) and old (10–12 days) flies were lysed in 300 μL malondialdehyde (MDA) lysis buffer and homogenised on a QIAshredder column (QIAGEN) at 13,000 g for 10 min. Homogenates were diluted 1:4 before measurement. Total protein concentration was quantified using the Pierce BCA protein assay kit (Thermo Scientific) on 25 μL of undiluted lysate, following the manufacturer’s instructions. Colorimetric measurements were performed at 532 nm using a Biotek Synergy 2 plate reader. All measurements were performed in duplicate, and lipid peroxidation levels were normalized against total protein content of the sample.

### Virus and bacterial infection

Fly stocks were cleared of *Wolbachia* and persistent virus infections as previously described^28^. After anesthesia with CO_2_, three to five-day-old flies were inoculated with virus by intrathoraxical injection with a Nanoject II injector (Drummond), or pricked with a needle dipped in a freshly grown bacteria pellet (OD_600_ = 100). Virus inocula contained 1,000 median tissue culture infectious doses (TCID_50_) of DCV and CrPV; 14,000 TCID_50_ of IIV-6; 3,000 TCID_50_ of FHV; and 2,000 TCID_50_ of DXV for all survival assays. An inoculum of 10,000 TCID_50_ of DCV was used in experiments in which transcriptional responses were analyzed. Flies were transferred to fresh food every 3 days and survival was assessed daily. Lethality on the first day was attributed to the injection procedure and excluded from the survival analysis. Unless noted otherwise, three pools of 10 to 15 flies were injected per condition with independent dilutions of virus stock.

### Virus titration

Viral titers were determined by end-point dilution, as previously described^28^.

### ***In vivo*** RNAi reporter assay

RNAi competency of adult flies was analyzed using a reporter assay, as described previously^21^. Briefly, three to five-day-old female flies were injected in the abdomen with a suspension containing lipofection reagent complexed with Firefly luciferase (Fluc) and Renilla luciferase (Rluc) reporter plasmids, along with Fluc specific or non-specific control (GFP) dsRNA. Fluc and Rluc activity was measured in fly homogenate, Fluc over Rluc ratios were calculated for each sample, and data are presented as fold silencing relative to the non-specific dsRNA control.

### qPCR analyses

DNA was isolated from flies with the QIAamp DNA blood mini kit (Qiagen) following the manufacturer’s instructions and 25 ng of DNA was used as input in the qPCR to quantify IIV-6 levels. RNA was isolated from flies using Isol-RNA lysis Agent (5-Prime). cDNA synthesis was performed on 1 μg of DNase I (Ambion)-treated RNA using TaqMan Reverse Transcription Reagents (Applied Biosystems) according to the manufacturers’ instructions. qPCR was performed using SYBR Green I Master reagents on a LightCycler 480 (Roche). The qPCR program was the following: 95 °C for 5 min, and 45 cycles of 95 °C for 5 s, 60 °C for 10 s, 72 °C for 20 s. Expression of the gene of interest was normalized to transcript levels of the housekeeping gene *Ribosomal Protein 49* (Rp49), and fold change was calculated using the ddCt method^31^. Primer sequences are provided in Supplementary Table 2.

### Plasmids

Insect expression plasmids pAc-tagRFP and pAc-tagEGFP were constructed by modifying pAc5.1-V5-His-A (Invitrogen) for expression of transgenes fused with GFP and RFP at the N-terminus. The full-length coding sequences of *Sgp* and *PMP34* were amplified from cDNA of adult *CnBw* flies, and cloned into pAc-tagRFP and pAc-tagEGFP, using SacI for *Sgp* and XbaI and SacI for *PMP34*. Primer sequences are provided in Supplementary Table 2.

### dsRNA synthesis

*In vitro* transcription using T7 RNA polymerase was performed on a PCR product flanked by T7 promoters. The reaction was incubated at 37 °C for 3 hours, followed by an incubation at 80 °C for 10 minutes and gradual cooling to room temperature. dsRNA was purified using the GenElute Mammalian Total RNA Miniprep Kit (Sigma) following the manufacturer’s instructions. Primer sequences are provided in Supplementary Table 2.

### Fluorescence microscopy

For subcellular localization of Sgroppino and PMP34, 2 × 10^5^ S2 cells (Invitrogen) per well were seeded in a 24-well plate. A day later, cells were transfected with 500 ng of pAc-RFP-Sgp and pAc-EGFP-PMP34, and, where applicable, 20 ng of dsRNA using Effectene transfection reagents (Qiagen) following the manufacturer’s instructions. Two days post-transfection, cells were resuspended and seeded on coverslips coated with 50 μL Concavalin A (0.5 mg/μL)^32^. Two hours later, samples were fixed with 4% paraformaldehyde for 20 minutes. The cover slips were then washed in PBS and permeabilized in 0.1% Triton X-100 in PBS for 15 minutes. Nuclei were stained with Hoechst 33342 reagent (Sigma) for 5 minutes (1:15,000 dilution from a stock concentration of 10 mg/mL in PBS/0.1% Triton), washed in PBS, and mounted with Mowiol 40–88 (Omnilabo). Pictures were taken on an Olympus FV1000 confocal microscope and processed using FIJI^33^. Colocalization was analyzed using ICYsoftware (version 1.9.6.0^34^). Briefly, the ICY spot detector^35^ was used to automatically detect puncta for the GFP and RFP signal within regions of interest (ROI, hand-delimited cells). Spots were detected with wavelet scales 2 and 3 at a 100% sensitivity. ROI for WAT (Wavelet Adaptive Threshold) calculation was used to correct for variation in background signal between the regions of interest. Per region of interest the distances between the center of green and red puncta were calculated to define colocalization (distance below 4 pixels). Colocalization was reported as percentage of RFP puncta that colocalized with GFP puncta.

### Statistical analysis

Unpaired two-tailed Student’s t-tests, as implemented in Graphpad Prism version 6, were used to compare differences in gene expression, viral RNA levels, and log-transformed viral titers. Survival assays were assessed using Kaplan-Meier analyses and log-rank tests, as implemented in SPSS Statistics (version 20, IBM). *P*-values below 0.05 were considered statistically significant.

## Results

### *Sgroppino* mutant flies are more sensitive to RNA virus infection

To identify novel genes induced upon virus infection, we previously analyzed the transcriptome of virus-infected flies, which were either mutant for the epigenetic regulator *G9a* or their wild-type controls, at 24 h post-infection (hpi)^21^. Components of the heat shock and Jak-Stat pathways were among the genes that were upregulated upon Drosophila C virus (DCV) infection; we analyzed their role in host defense previously^21,25^. Amongst the genes with the highest induction in *G9a* mutants was the uncharacterized gene *CG13091*, which we later named *Sgroppino* (*Sgp*) (Supplementary Table 1).

**Figure 1:**
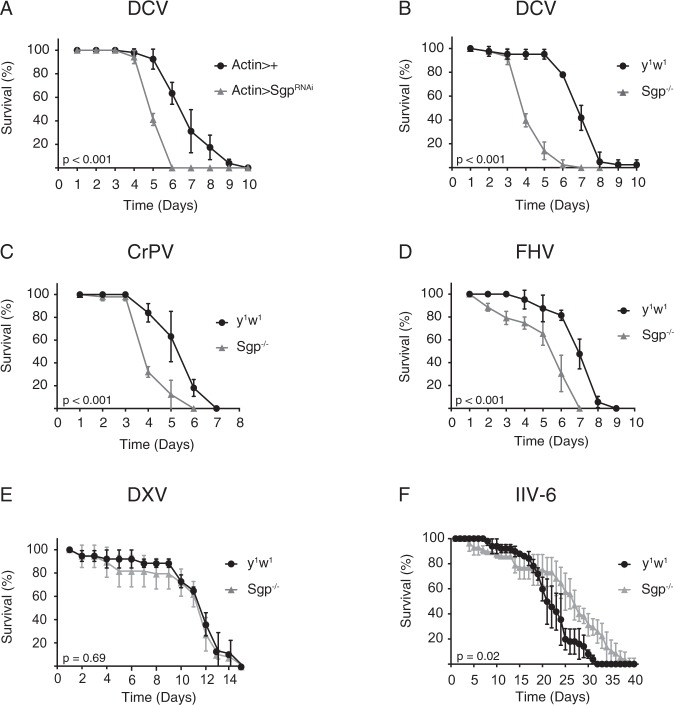
*Sgroppino* mutants are hypersensitive to RNA virus infection. (**A**) Survival upon DCV infection of flies ubiquitously expressing an RNAi-inducing hairpin targeting *Sgp*. The ubiquitous Actin driver line (*Actin-Gal4*) was used to drive expression of the transcription factor Gal4, which binds the Upstream Activating Sequence to induce expression a short hairpin RNA targeting *Sgp* (*UAS-Sgp*^RNAi^). Flies expressing the *Actin-Gal4* driver, but not the UAS responder (*Actin-Gal4*>+), were included as controls. (**B–F**) Survival of wild-type (*y*^1^*w*^1^) and *Sgp*^−/−^ mutant flies upon (**B**) DCV, (**C**) CrPV, (**D**) FHV, (**E**) DXV and (**F**) IIV-6 infection. Data represent means and s.d. of three biological replicates of at least 15 female flies for each genotype.

To determine whether *Sgroppino* is important in host defense, we reduced *Sgp* expression by expression of an RNAi-inducing hairpin RNA (*Sgp*^RNAi^) under control of the ubiquitous *Actin*-*Gal4* driver and monitored survival rates upon viral challenge. Upon infection with DCV, a positive-sense RNA virus from the *Dicistroviridae* family, *Sgp*^RNAi^ flies exhibited lower survival rates than control flies expressing the *Actin-Gal4* driver only (Fig. 1A; mean survival = 5.3 and 7.0 days, respectively; *P* < 0.001). We used reverse transcription followed by quantitative PCR (RT-qPCR) to confirm that *Sgp* knock-down was efficient, and found that *Sgp* mRNA levels were reduced by 80% in male and female flies (Supplementary Fig. 1A).

Next, we sought to confirm this observation using a mutant fly line (*Sgp*^EY06744^) containing a P{EPgy2} transposon insertion in the 3′-untranslated region of the *Sgp* gene^26^ (Supplementary Fig. 1B). *Sgp* expression was reduced by 96% in these flies compared to wildtype control flies, and the insertion affected expression levels of both isoforms (Supplementary Fig. 1C). For the remainder of our study we used this hypomorphic mutant, which we refer to as *Sgp*^−/−^. *Sgroppino-*deficient flies had no obvious defects in development and exhibited a similar lifespan as wild-type flies under standard laboratory conditions (Supplementary Fig. 1D). Upon challenge with DCV, *Sgp* mutant flies had a reduced survival time compared to the wild-type control (*y*^1^*w*^1^) flies (Fig. 1B; mean survival = 4.4 and 7.1 days, respectively; *P* < 0.001), confirming the phenotype of *Sgp*^RNAi^ flies (Fig. 1A). We verified that mock infection did not affect survival rates as a result of injury-associated stress and mortality (Supplementary Fig. 2A). Moreover, abiotic stresses, such as heat shock and starvation did not trigger premature mortality (Supplementary Fig. 2B,C), excluding a broad sensitivity to various stressors.

We next challenged *Sgp* mutants with another dicistrovirus, cricket paralysis virus (CrPV). As for DCV, infected mutant flies succumbed earlier to CrPV challenge than wild-type flies (Fig. 1C; mean survival = 4.4 and 5.6 days, respectively; *P* < 0.001). Hypersensitivity to viral infection was not sex-dependent, as male *Sgp* mutant flies also had reduced survival rates upon DCV (Supplementary Fig. 2D; mean survival = 5.3 and 3.2 days; *P* < 0.001) and CrPV infection (Supplementary Fig. 2E Fig; mean survival = 4.0 and 6.0 days; *P* < 0.001). Next, we evaluated survival rates upon infection with two other RNA viruses: Flock House virus (FHV), a positive sense RNA virus from the *Nodaviridae* family, and Drosophila X virus (DXV), a double stranded RNA virus from the *Birnaviridae* family. Mean survival time was 6.7 days for wild-type flies upon FHV infection, which was significantly reduced to 5.3 in *Sgp* mutant flies (Fig. 1D; *P* < 0.001). Conversely, no difference in survival was observed upon DXV infection of *Sgp* mutant flies compared to control flies (Fig. 1E). Potential explanations for dissimilarities in susceptibility to different viruses could be virus-specific effects, differences in viral tropism and tissue-specific expression of *Sgp*. Indeed, we found that *Sgp* was expressed at 8-fold higher levels in the fat body than in the whole body (Supplementary Fig. 2F; *P* = 0.02).

To test whether *Sgp* mutants were also more sensitive to DNA virus infection, we challenged flies with invertebrate iridescent virus 6 (IIV-6). In contrast to our observations with RNA viruses, survival rates of *Sgp* mutants were slightly higher, with mean survival times of 16.5 days for wild-type flies and 19.1 days for *Sgp* mutants (Fig. 1F; *P* = 0.02). Together, our results indicate that *Sgroppino* mutants are hypersensitive to infections with several RNA viruses, but not a DNA virus.

### Higher DCV genomic RNA replication in *Sgroppino* mutant flies

To determine whether hypersensitivity to virus infection was accompanied by higher viral replication, we determined infectious titers using endpoint dilution assays and viral RNA levels by RT-qPCR over a 2-day time course after challenge with the panel of viruses of Fig. 1.

**Figure 2:**
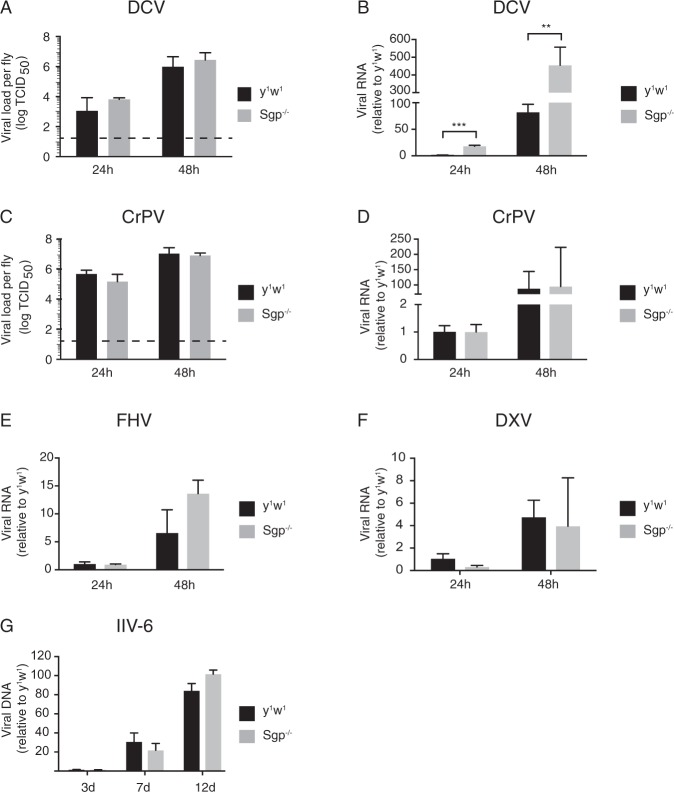
Higher DCV RNA levels in *Sgroppino* mutants. (**A**,**C**) Viral titers in wild-type and *Sgp* mutant flies inoculated with (**A**) DCV and (**C**) CrPV at 24 and 48 hpi. The dashed line represents the detection limit of the titration. (**B,D**–**G**) Viral RNA or DNA levels measured by (RT-)qPCR in wild-type and *Sgp* mutant flies infected with (**B**) DCV, (**D**) CrPV, (**E**) FHV, (**F**) DXV and (**G**) IIV-6. Viral RNA/DNA levels were normalized against transcript levels of the housekeeping gene *Ribosomal Protein 49* and presented as fold change relative to wild-type flies at 24 hpi (**B**,**D**–**F**) or 3 days post-infection (**G**). *P < 0.05, **P < 0.01, ***P < 0.001 (Student’s t-test). Data represent (**A,C**) mean and s.d. of three independent experiments, each consisting of three replicates of at least 5 female flies for each genotype, or (**B**,**D–G**) means and s.d. of three biological replicates of at least 15 female flies for each genotype.

Upon DCV infection, we observed a ~6-fold increase in viral titers in *Sgp* mutants relative to wild-type controls at 24 hpi and a less pronounced, 3-fold difference at 48 hpi, but these differences did not reach statistical significance (Fig. 2A). Next, we measured DCV RNA levels and observed an 18-fold increase in DCV levels at 24 hpi (*P* < 0.001) and a 5.5-fold increase at 48 hpi in *Sgp* mutants relative to wild-type flies (*P* < 0.01, Fig. 2B). In contrast, upon CrPV infection, no difference was found for viral titers and viral RNA levels, both at 24 and 48 hpi (Fig. 2C,D). Similarly, no significant increase in viral RNA levels could be detected in *Sgp*^−/−^ flies upon FHV and DXV infection over the 2 days following infection (Fig. 2E,F). Finally, we measured viral DNA levels of IIV-6, and found no significant differences between *Sgp* mutant flies and wild-type controls at any of the time points analyzed (3, 7 and 12 dpi, Fig. 2G). Together, these results indicate that the hypersensitivity of *Sgroppino* mutant flies to RNA virus infection is associate with higher RNA replication of Drosophila C virus, but not of the other viruses.

### RNA interference and canonical immune pathways are intact in *Sgroppino*-deficient flies

**Figure 3:**
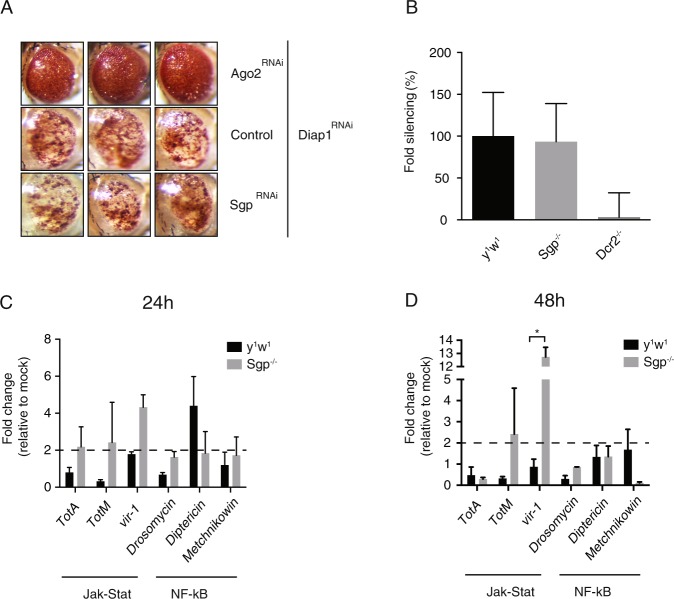
RNAi and canonical immune pathways are functional in *Sgroppino* mutants. (**A**) Eye phenotype of 5 to 7-day-old flies expressing a hairpin RNA targeting the Death-associated inhibitor of apoptosis *Diap1* (*Diap1*^RNAi^), combined with either a hairpin RNA targeting *Ago2* (*Ago2*^RNAi^), a hairpin targeting *Sgp* (*Sgp*^RNAi^), or the genetic background of the *Sgp*^RNAi^ line (control). Wild-type eye phenotype was observed in flies not expressing the *Diap1*^RNAi^ transgene from the same cross. Three representative images are shown for each genotype. (**B**) *In vivo* RNAi reporter assay. Firefly (Fluc) and Renilla (Ren) luciferase reporter plasmids were co-transfected with Fluc specific dsRNA or non-specific control dsRNA in *Sgp* and *Dcr2*^−/−^ mutant flies and wild-type control flies (*y*^1^*w*^1^). Fold silencing by Fluc dsRNA relative to control dsRNA was calculated and presented as percentage of wild-type controls. Data are means and s.d. of three independent pools of 5 female flies for each genotype. (**C,D**) Expression of immune genes at (**C**) 24 and (**D**) 48 hours after DCV infection (inoculum of 10,000 TCID_50_) determined by RT-qPCR in wild-type or *Sgp* mutant flies. Expression of the indicated genes was normalized to transcript levels of the housekeeping gene *Ribosomal Protein 49* and expressed as fold change relative to mock infection (Tris buffer). Data are means and s.d. of three independent pools of 10 female flies for each genotype. *P < 0.05 (Student’s t-test).

To characterize the mechanism underlying the hypersensitivity of *Sgp* mutant flies to virus infection, we asked whether canonical antiviral defenses were functional in those mutants. RNAi is one of the main antiviral immune pathways in *Drosophila*^12,13^. To test RNAi functionality, we used an *in vivo* sensor assay^21^ that is based on an RNAi-inducing hairpin RNA that silences the inhibitor of apoptosis *Death-associated inhibitor of apoptosis 1*, also known as *thread* (*Diap1*^RNAi^)^27,36^. When driven specifically in the eye using the *GMR-Gal4* driver, expression of *Diap1*^RNAi^ triggers severe apoptosis in the developing eye, characterized by loss of pigmentation and reduced size (Fig. 3A). *Argonaute 2* (*AGO2*) mutant flies missing the central catalytic component of the RNAi pathway do not exhibit this phenotype, demonstrating its full dependence on the RNAi pathway^27,36^. We confirmed this here, as concomitant expression of *Diap1*^RNAi^ and *Ago2*^RNAi^ hairpins did not induce the eye phenotype (Fig. 3A). Simultaneous expression of *Diap1*^RNAi^ and *Sgp*^RNAi^ hairpins, however, induced a similar eye phenotype as *Diap1*^RNAi^ in control flies (Fig. 3A), suggesting that the RNAi pathway is functional in *Sgroppino*-deficient flies.

To confirm this observation, we used a luciferase-based RNAi sensor assay to assess RNAi efficiency in *Sgp*^−/−^ mutant flies, as described previously^25,36^. Silencing efficiency was measured in fly lysates, collected 3 days after *in vivo* transfection with Firefly (Fluc) and *Renilla* luciferase reporter plasmids together with either Fluc-specific dsRNA or control dsRNA. The strong reduction of silencing activity in *Dicer-2* null mutants, compared to their wild-type controls (*y*^1^*w*^1^), demonstrates that silencing of Fluc expression was RNAi-dependent (Fig. 3B). Silencing efficiencies in *Sgp*^−/−^ mutant flies and controls were similar, confirming that RNAi is fully functional in *Sgp*^−/−^ mutant flies (Fig. 3B).

In addition to the RNAi pathway, viral infection has been shown to activate several immune pathways^15,37^. For example, the Jak-Stat pathway controls expression of genes encoding the stress-related proteins Turandot A and M (*TotA* and *TotM*) and the infection-induced gene *virus induced RNA-1* (*vir-1*)^14,16^. The NF-κB-related Toll and Imd pathways, regulate expression of genes encoding antimicrobial peptides, such as Drosomycin, Metchnikowin, and Diptericin, which are secreted by the fat body upon bacterial challenge and in some cases upon viral infections^17–19^. To test whether these signaling cascades are functional in *Sgp* mutant flies, we monitored expression of these downstream genes by RT-qPCR, at 24 and 48 hours after DCV infection (Fig. 3C,D).

No significant induction of Jak-Stat or NF-κB-dependent genes was detected at 24 hpi with DCV in wild-type or *Sgp* mutant flies (Fig. 3C). Amongst the genes analyzed, we observed the highest induction (4-fold) for *vir-1* in *Sgp* mutants. However, for none of the genes a significant difference was observed between mutant flies and controls at 24 hpi (Fig. 3C). At 48 hpi, expression of *vir-1* was strongly increased in *Sgp* mutants (12.7-fold), whereas it was not induced in wild-type flies (0.8-fold, *P* < 0.05; Fig. 3D). This is most likely due to higher DCV levels in *Sgp* mutants (Fig. 2A,B). We noted that induction of these canonical Jak-Stat dependent genes was consistently lower than previously reported with the same virus dose^21^, which is most likely due to the different genetic background. For the other Jak-Stat or NF-κB regulated genes, we observed only low induction upon infection, and, more importantly, no significant difference between wild-type and *Sgp* mutant flies. We also verified that constitutive expression levels of Jak-Stat dependent genes were similar in wild-type and *Sgp* mutant flies (Supplementary Fig. 3A). Since only low expression of NF-κB-dependent genes was expected upon systemic viral challenge (reported previously in^14,17,21,38^), we also measured the expression of *Drosomycin*, *Metchnikowin*, *Drosocin*, *Diptericin B, Immune induced 1*, and *Cecropin A2* at 6 and 24 hpi with Gram positive (*Micrococcus luteus*) and Gram negative bacteria (*Erwinia caratovora caratovora 15*, *Ecc 15*) (Supplementary Fig. 3B–G). Overall, no significant differences in AMP induction levels were observed between *Sgp* mutants and control flies, at both time points and upon both challenges. Taken together, these date indicate that canonical antiviral defense mechanisms are intact in *Sgroppino* mutant flies.

### Sgroppino partially localizes to peroxisomes

**Figure 4:**
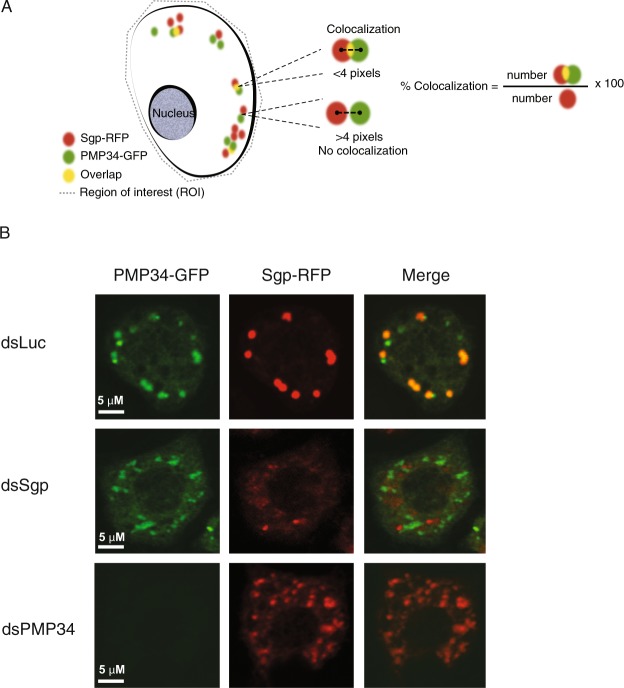
(A) Sgroppino localizes to peroxisomes. Schematic representation of the approach used to quantify colocalization between Sgroppino and Peroxisomal Membrane Protein (PMP34). (**B**) Localization of RFP-tagged Sgroppino and GFP-tagged PMP34 in *Drosophila* S2 cells. Expression plasmids were co-transfected with dsRNA targeting *Sgp*, *PMP34*, or, as a non-targeting control, *Luciferase* (dsLuc), and cells were fixed and processed two days later. Images were obtained by confocal microscopy.

To obtain more insights into the function of Sgroppino, we analyzed its intracellular localization in *Drosophila* S2 cells. In a previously published *in silico* analysis, *Sgroppino* was predicted as one of 17 *Drosophila* orthologs of the human fatty acyl-CoA reductase (FAR-1) gene (although not the closest ortholog). FAR-1 transforms fatty acyl CoA into fatty alcohol within the ether lipid synthesis pathway in peroxisomes^39^. To determine whether Sgroppino localizes to peroxisomes in *Drosophila*, we expressed *Sgp* fused to Red Fluorescent Protein (RFP) at its N-terminus from an Actin promoter-driven expression plasmid. As a marker for peroxisomes, we used an expression vector encoding N-terminally GFP-tagged Peroxisomal Membrane Protein 34 (PMP34), which contains a peroxisome membrane targeting signal and six transmembrane domains and localizes in the peroxisomal membrane^40^. To control for specificity and to determine whether *Sgp* knock-down affects peroxisome integrity, we cotransfected dsRNA targeting *Sgp*, *PMP34*, or, as a negative control, *luciferase*. Upon transfection with control dsRNA, we observed punctate cytoplasmic GFP staining demonstrating that PMP34-GFP localizes to peroxisomes, as expected (Fig. 4). Strikingly, Sgroppino-RFP repeatedly localized to the similar puncta, and the merged images reveal partial colocalization of Sgroppino and PMP34. We quantified colocalization in a total of 108 cells, defined as a distance below 4 pixels between the centers of RFP and GFP-positive puncta (Fig. 4A). We found that 50,4% of the Sgroppino-RFP colocalized with peroxisomal PMP34-GFP puncta, indicating that Sgroppino localizes, at least partly, to peroxisomes. As expected, RFP or GFP signals were strongly reduced upon knock-down of *Sgp* and *PMP34*, respectively, demonstrating the specificity of the fluorescent signal (Fig. 4B). Moreover, *Sgp* knock-down did not affect the distribution or density of PMP34-GFP puncta, suggesting that *Sgp* is not required for peroxisome biogenesis or integrity. These results demonstrate that *Sgp* partially localizes to peroxisomes in *Drosophila* S2 cells.

### Sgroppino mutant flies have a defect in lipid metabolism

**Figure 5:**
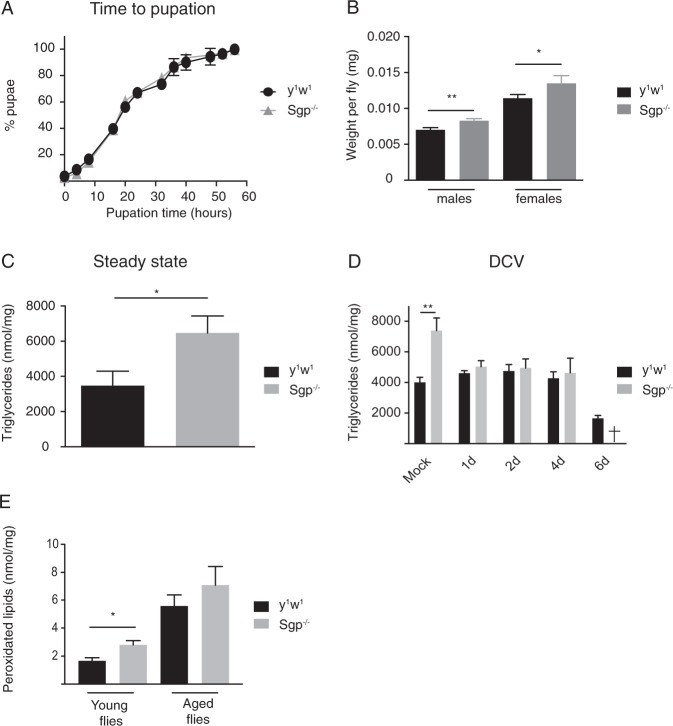
*Sgroppino*-deficiency causes weight increase and accumulation of fat. (**A**) Time to pupation of wild-type and *Sgp* mutant flies. Fifty eggs were incubated on standard cornmeal-agar media at 25 °C, and monitored for the appearance of pupae at least twice a day. The 0 h time point corresponds to the appearance of the first pupae, which was identical for wild-type and mutant flies. (**B**) Weight of female and male wild-type and *Sgp* mutant flies. Three to five-day-old flies were weighed in groups of 10 on a precision scale. (**C**,**D**) Levels of triglycerides in 3 to 5-day-old female wild-type and *Sgp* mutant flies at steady-state (**C**) or upon DCV infection (**D**). All DCV infected *Sgp* mutant flies had died at 6 dpi. (**E**) Levels of peroxidated lipids in young (2–4 day old) or aged flies (10–12 day old). Data represent mean and s.d. of three biological replicates of (**A**) 50 eggs, (**B**) 10 flies, (**C**,**D**) 2 flies, and (**E**) 20 to 40 flies for each genotype. Mock infection was performed with Tris Buffer, and harvested at day 2 (**D**). Student’s t-tests were used to compare the differences in weight and triglycerides (**P < 0.01, ***P < 0.001).

Peroxisomes are intracellular organelles that are important for lipid metabolism, including ether lipid biosynthesis, α-oxidation of branched chain fatty acids, and β-oxidation of fatty acids. During β-oxidation, reactive oxygen species (ROS) are generated and peroxisomes contain enzymes (oxidases and catalases) that regulate oxidative stress^39^. Thus, we sought to determine whether *Sgroppino* mutants had major defects in metabolism or growth. Pupation is a highly regulated process in *Drosophila* that depends amongst others on hormonal signaling, circadian clock, and weight^41,42^. To test how the time to pupation of *Sgroppino* mutants compares to wild-type flies, we analyzed the formation of pupae in vials in which the same number of embryos had been placed. However, we found no significant difference in pupae formation between wild-type and *Sgp* mutant flies, suggesting that *Sgroppino* deficiency does not impact the timing of larval growth and the transition from the larval to the pupal stage (Fig. 5A).

We next determined the weight of adult flies, and noticed a significant increase in the weight of *Sgp* mutants compared to wild-type flies, both in males and females (Fig. 5B). The increased weight of adults was even noticeable visually, with *Sgp* mutants having larger abdomens than wild-type flies. In agreement, we observed that, after removal of the digestive track and reproductive system, a larger mass of fat body tissue remained loosely attached to the abdominal carcass (as illustrated in Supplementary Fig. 4). It formed large oleaginous droplets and appeared white (which reminded us of the *Sgroppino* cocktail). The *Drosophila* fat body is a multi-functional organ involved, for instance, in the storage of fat, and the secretion of humoral immune factors and endocrine mediators^43,44^. In this organ, adipocytes store energy in the form of glycogen and triglycerides, which may be recruited in response to energy demands of the insect. It was previously demonstrated that high calorie diet leads to the storage of excess triglycerides in large droplets in the fat body^45^. Our visual observation (Supplementary Fig. 5), together with the increased weight of *Sgp* mutants (Fig. 5B) prompted us to quantify triglyceride levels. We found that *Sgp* mutant flies contained significantly higher amounts of triglycerides than wild-type flies at steady-state levels (Fig. 5C). Upon mock infection, triglyceride levels remained significantly different, but upon challenge with DCV, triglyceride levels were similar between *Spg* mutants and wild-type flies at 1, 2, and 4 dpi (Fig. 5D).

Lipid peroxidation is defined as the degradation of lipids through oxidation of long chain fatty acids, which occurs partly in peroxisomes^46^. Quantification of malondialdehyde (MDA), a byproduct of lipid peroxidation, is quantifiable with a colorimetric assay and can be used as a proxy for lipid peroxidation levels, and thus, peroxisomal lipid degradation function. Using this assay, we measured levels of lipid peroxidation in young (2–4 day old) and aged (10–12 day old) flies, and found a significant increase in lipid peroxidation in young *Spg*^−/−^ flies compared to control flies of the same age (Fig. 5E). Together, these observations suggest that *Sgp* mutants have a defect in lipid metabolism which is likely related to Sgroppino function in peroxisomes.

### Peroxisomes are required for antiviral host defense

**Figure 6:**
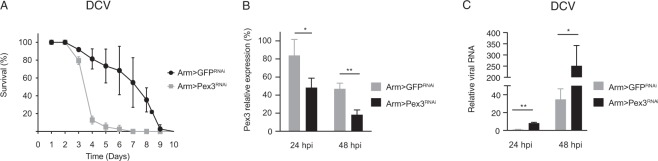
Peroxisomes are required for host defense to DCV infection. (**A**) Survival upon DCV infection of flies expressing an RNAi-inducing hairpin targeting *Pex3* or *GFP*. The ubiquitous *armadillo* driver (*arm-Gal4*) was used to drive expression of the transcription factor Gal4, which binds to the Upstream Activating Sequence to induce expression a short hairpin RNA targeting *Pex3* (Arm > Pex3^RNAi^) or *GFP* (Arm > GFP^RNAi^). (**B**) *Pex3* expression levels and (**C**) viral RNA levels upon DCV infection of *Pex3*^RNAi^ flies. Expression of *Pex3* was normalized to transcript levels of the housekeeping gene *Ribosomal Protein 49* and expressed as percentage of Arm >+ controls. Expression of viral RNA levels was normalized to the housekeeping gene *Ribosomal Protein 49* and expressed as fold change relative to the *GFP*^RNAi^ control flies at 24 hpi. Data in panels A–C were collected in parallel; knock-down efficiencies in (**B**) thus apply to the experiments in (**A**,**C**). Data represent means and s.d. of three independent pools of (**A,C**) 5 or (**B**) 15 female flies for each genotype. Student’s t-tests were used to compare the differences in *Pex3* or DCV RNA levels (*P < 0.05, **P < 0.01).

As Sgroppino is important for antiviral defense and localizes to peroxisomes, we asked whether these organelles are necessary for host defense. To this end, we used RNAi to reduce expression of *Pex3*, an essential factor for *de novo* peroxisome biogenesis and function^47^. As previously shown, elimination or strong reduction of the number of peroxisomes is developmentally lethal^47^. To achieve a non-lethal reduction in the number of peroxisomes, we induced ubiquitous knock-down of *Pex3* using the *armadillo-Gal4* driver. We challenged *Pex3-*deficient flies (Arm > Pex3^RNAi^) and, as control, flies expressing a GFP^RNAi^ hairpin (Arm > GFP^RNAi^) with DCV and monitored survival rates. The mean survival time of *Pex3* knock-down flies was 4 days, whereas it was 7 days for Arm > GFP^RNAi^ control flies (*P* < 0.001) (Fig. 6A). We performed mock infections to verify that the early mortality observed in *Pex3-*deficient flies was indeed due to DCV infection (Supplementary Fig. 5). We used RT-qPCR to confirm that *Pex3* expression was modestly, but significantly reduced at 24 and 48 hpi (1.8 and 2.6 fold reduction, respectively, Fig. 6B). Under these conditions, viral RNA levels were increased 8.2 and 7.5-fold in *Pex3*-deficient flies relative to the controls at 24 and 48 hpi, respectively (Fig. 6C). We thus conclude that peroxisomes are necessary for effective host response to DCV infection, the direct involvement of Sgp in that function remains to be established.

## Discussion

Although several mechanisms for antiviral host defense have been discovered in *Drosophila* over the past years, our knowledge remains incomplete. Here, we propose *Sgroppino* as a player in the antiviral host defense. *Sgroppino*-deficient flies are hypersensitive to infection with a panel of single-stranded RNA viruses infection, of which DCV replicates to higher levels. *Sgroppino* localizes to peroxisomes, and partial depletion of *Pex3*, a peroxisome biogenesis factor, increases sensitivity of adult flies to DCV infection, accompanied by an increase in virus replication. Overall, our data indicate that *Sgroppino* participates in the host response to viral infection, conceivably through its function in lipid metabolism within peroxisomes.

The mechanism by which *Sgroppino* affects virus infection remains to be defined. Sgroppino is one of 17 *Drosophila* orthologs of fatty acyl-CoA reductases that are predicted to reduce fatty acids into fatty alcohols, an intermediate in the biosynthesis of waxes and ether lipids^39^. Ether lipids are thought to contribute to a decrease in membrane fluidity, and might act as scavengers for reactive oxygen species to avoid the oxidation of other exposed membrane lipids^48^. At the subcellular level, ether lipid deficiency alters cholesterol distribution, resulting in cholesterol accumulation in endosomal and lysosomal compartments, and causing structural changes in the ER and Golgi apparatus^49–51^. As both cholesterol and intracellular membranous networks are exploited by viruses for their replication^2,5,52–54^, it is possible that the observed dysregulation in lipid metabolism in *Sgroppino*-deficient flies generates an environment that is favorable for virus replication.

Strikingly, *Sgp* mutant flies harbor higher levels of virus when infected with DCV, but not other single-stranded RNA viruses (CrPV and FHV), even though higher mortality rates were observed for all three viruses. *Sgroppino* is expressed at high levels in the adult fat body, according to our data (Supplementary Fig. 2F) and data from FlyAtlas^55^. The abdominal fat body supports high DCV replication in adult flies^56^, which may explain the more pronounced phenotype and higher DCV RNA load of *Sgp* mutants. We cannot exclude that hypersensitivity to CrPV and FHV infection is caused by tissue-specific differences in viral replication that are below the sensitivity threshold of our assays in entire flies. Another possibility is that *Sgroppino* also influences tolerance to infection, which is the ability of a host to endure an infection^28,57–59^.

The fat body plays an essential role in the storage and release of energy. Fatty acids are stored in the form of triglycerides along with other neutral lipids in lipid droplets of adipocytes. Sgroppino is expected to consume fatty acids after conversion into fatty acyl-CoA for the production of ether-linked lipids, one of the major metabolic pathways in peroxisomes^39^. Intriguingly, studies in yeast revealed that peroxisomes form extensive physical contacts with lipid droplets, coupling metabolic pathways of both compartments. In the absence of Pex5, which leads to peroxisomal malfunction, lipids that fail to be oxidized accumulate in the cytoplasm^60^. It is possible that reduced consumption of lipids in *Sgp* mutant flies trigger the accumulation of unprocessed lipid inclusions in cells, explaining the increased mass of fat tissue in the fly abdomen.

Peroxisomes are mostly studied for their metabolic functions, but were recently found to play a role in immunity. In mammals, peroxisomes have been proposed as platforms for antiviral signal transduction, as had previously been reported for mitochondria^61,62^. RIG-I like receptors (RLR) can signal via MAVS on peroxisomes to drive expression of type III interferons, which have tissue-specific functions in antiviral immunity^63^. However, we did not find obvious defects in Jak-Stat or NF-κB signaling, suggesting that the *Sgroppino* phenotype is not caused by defects in canonical immune pathways. A recent study showed that peroxisomes play an essential role in phagocytosis of bacteria in *Drosophila* and mouse macrophages^64^. As phagocytosis also contributes to virus-specific immune responses in *Drosophila*^38^, it is possible that defects in phagocytic processes explains the hypersensitivity of *Sgp* mutants to viral infection.

Human diseases linked to peroxisome dysfunction, such as Zellweger Syndrome, are rare and difficult to treat. Recent development of fly models for peroxisomal defects^39,47,65,66^ offer great promise to study peroxisomal functions in metabolism, and, as our results suggest, immunity.

## Supplementary information

Supplemental Data

## Data Availability

The datasets generated and/or analyzed during the current study are available from the corresponding author on reasonable request.

## References

[1] King, A. M. Q., Lefkowitz, E., Adams, M. J. & Carstens, E. B. *Virus Taxonomy: Ninth Report of the International Committee on Taxonomy of Viruses*. (Elsevier Science, 2011).

[2] Inoue T, Tsai B (2013). How viruses use the endoplasmic reticulum for entry, replication, and assembly. Cold Spring Harb. Perspect. Biol..

[3] Mercer J, Schelhaas M, Helenius A (2010). Virus entry by endocytosis. Annu. Rev. Biochem..

[4] Miller DJ, Schwartz MD, Ahlquist P (2001). Flock house virus RNA replicates on outer mitochondrial membranes in Drosophila cells. J. Virol..

[5] Romero-Brey I, Bartenschlager R (2014). Membranous replication factories induced by plus-strand RNA viruses. Viruses.

[6] Tolonen N, Doglio L, Schleich S (2001). & Krijnse Locker, J. Vaccinia virus DNA replication occurs in endoplasmic reticulum-enclosed cytoplasmic mini-nuclei. Mol. Biol. Cell.

[7] Chow J, Franz KM, Kagan JC (2015). PRRs are watching you: Localization of innate sensing and signaling regulators. Virology.

[8] Kumar H, Kawai T, Akira S (2011). Pathogen recognition by the innate immune system. Int. Rev. Immunol..

[9] Hoffmann JA (2003). The immune response of Drosophila. Nature.

[10] Buchon N, Silverman N, Cherry S (2014). Immunity in Drosophila melanogaster - from microbial recognition to whole-organism physiology. Nat. Rev. Immunol..

[11] Chambers, M. C. & Schneider, D. S. Pioneering immunology: insect style. *Curr. Opin. Immunol*., 1–5, 10.1016/j.coi.2011.11.003 (2011). 10.1016/j.coi.2011.11.003 22188798

[12] Bronkhorst AW, van Rij RP (2014). The long and short of antiviral defense: small RNA-based immunity in insects. Curr Opin Virol.

[13] Kemp C, Imler J-L (2009). Antiviral immunity in drosophila. Curr. Opin. Immunol..

[14] Kemp C (2013). Broad RNA interference-mediated antiviral immunity and virus-specific inducible responses in Drosophila. J. Immunol..

[15] Merkling SH, van Rij RP (2013). Beyond RNAi: antiviral defense strategies in Drosophila and mosquito. J. Insect Physiol..

[16] Dostert C (2005). The Jak-STAT signaling pathway is required but not sufficient for the antiviral response of drosophila. Nat. Immunol..

[17] Costa A, Jan E, Sarnow P, Schneider D (2009). The Imd pathway is involved in antiviral immune responses in Drosophila. Plos One.

[18] Avadhanula V, Weasner BP, Hardy GG, Kumar JP, Hardy RW (2009). A novel system for the launch of alphavirus RNA synthesis reveals a role for the Imd pathway in arthropod antiviral response. PLoS Pathog..

[19] Zambon Ra, Nandakumar M, Vakharia VN, Wu LP (2005). The Toll pathway is important for an antiviral response in Drosophila. Proc. Natl. Acad. Sci. USA.

[20] Ferreira ÁG (2014). The Toll-Dorsal Pathway Is Required for Resistance to Viral Oral Infection in Drosophila. PLoS Pathog..

[21] Merkling SH (2015). The epigenetic regulator g9a mediates tolerance to RNA virus infection in Drosophila. PLoS Pathog..

[22] Moy RH (2014). Antiviral Autophagy Restricts Rift Valley Fever Virus Infection and Is Conserved from Flies to Mammals. Immunity.

[23] Nakamoto M (2012). Virus recognition by Toll-7 activates antiviral autophagy in Drosophila. Immunity.

[24] Shelly S, Lukinova N, Bambina S, Berman A, Cherry S (2009). Autophagy is an essential component of Drosophila immunity against vesicular stomatitis virus. Immunity.

[25] Merkling SH (2015). The heat shock response restricts virus infection in Drosophila. Sci Rep.

[26] Bellen HJ (2004). The BDGP gene disruption project: single transposon insertions associated with 40% of Drosophila genes. Genetics.

[27] Meyer WJ (2006). Overlapping functions of argonaute proteins in patterning and morphogenesis of Drosophila embryos. Plos Genet..

[28] Merkling SH, van Rij RP (2015). Analysis of resistance and tolerance to virus infection in Drosophila. Nat. Protoc..

[29] Magwire MM (2012). Genome-wide association studies reveal a simple genetic basis of resistance to naturally coevolving viruses in Drosophila melanogaster. Plos Genet..

[30] Smith EM (2007). Feeding Drosophila a biotin-deficient diet for multiple generations increases stress resistance and lifespan and alters gene expression and histone biotinylation patterns. J. Nutr..

[31] Livak KJ, Schmittgen TD (2001). Analysis of Relative Gene Expression Data Using Real-Time Quantitative PCR and the 2−ΔΔCT Method. Methods.

[32] Rogers SL, Rogers GC (2008). Culture of Drosophila S2 cells and their use for RNAi-mediated loss-of-function studies and immunofluorescence microscopy. Nat. Protoc..

[33] Schindelin J (2012). Fiji: an open-source platform for biological-image analysis. Nat. Methods.

[34] de Chaumont F (2012). Icy: an open bioimage informatics platform for extended reproducible research. Nat. Methods.

[35] Olivo-Marin J-C (2002). Extraction of spots in biological images using multiscale products. Pattern Recognition.

[36] van Mierlo JT (2012). Convergent evolution of argonaute-2 slicer antagonism in two distinct insect RNA viruses. Plos Pathog..

[37] Lamiable O, Imler JL (2014). Induced antiviral innate immunity in Drosophila. Curr. Opin. Microbiol..

[38] Lamiable O (2016). Analysis of the Contribution of Hemocytes and Autophagy to Drosophila Antiviral Immunity. J. Virol..

[39] Faust JE, Verma A, Peng C, McNew JA (2012). An inventory of peroxisomal proteins and pathways in Drosophila melanogaster. Traffic.

[40] Sugiura A, Mattie S, Prudent J, McBride HM (2017). Newly born peroxisomes are a hybrid of mitochondrial and ER-derived pre-peroxisomes. Nature.

[41] De Moed GH, Kruitwagen CLJJ, De Jong G, Scharloo W (1999). Critical weight for the induction of pupariation in Drosophila melanogaster: genetic and environmental variation. J. Evol. Biol..

[42] Di Cara F, King-Jones K (2013). How clocks and hormones act in concert to control the timing of insect development. Curr. Top. Dev. Biol..

[43] Li, S., Yu, X. & Feng, Q. Fat Body Biology in the Last Decade. *Annu. Rev. Entomol*., 10.1146/annurev-ento-011118-112007 (2018). 10.1146/annurev-ento-011118-112007 30312553

[44] Arrese EL, Soulages JL (2010). Insect fat body: energy, metabolism, and regulation. Annu. Rev. Entomol..

[45] Musselman LP (2013). Role of fat body lipogenesis in protection against the effects of caloric overload in Drosophila. J. Biol. Chem..

[46] Ayala A, Munoz MF, Arguelles S (2014). Lipid peroxidation: production, metabolism, and signaling mechanisms of malondialdehyde and 4-hydroxy-2-nonenal. Oxid. Med. Cell. Longev..

[47] Faust JE (2014). Peroxisomes are required for lipid metabolism and muscle function in Drosophila melanogaster. Plos One.

[48] Braverman NE, Moser AB (2012). Functions of plasmalogen lipids in health and disease. Biochim. Biophys. Acta.

[49] Gorgas K, Teigler A, Komljenovic D, Just WW (2006). The ether lipid-deficient mouse: tracking down plasmalogen functions. Biochim. Biophys. Acta.

[50] Thai TP (2001). Impaired membrane traffic in defective ether lipid biosynthesis. Hum. Mol. Genet..

[51] Schedin S, Sindelar PJ, Pentchev P, Brunk U, Dallner G (1997). Peroxisomal impairment in Niemann-Pick type C disease. J. Biol. Chem..

[52] Ilnytska O (2013). Enteroviruses harness the cellular endocytic machinery to remodel the host cell cholesterol landscape for effective viral replication. Cell Host Microbe.

[53] Rothwell C (2009). Cholesterol biosynthesis modulation regulates dengue viral replication. Virology.

[54] Liefhebber JM, Hague CV, Zhang Q, Wakelam MJ, McLauchlan J (2014). Modulation of triglyceride and cholesterol ester synthesis impairs assembly of infectious hepatitis C virus. J. Biol. Chem..

[55] Robinson SW, Herzyk P, Dow JA, Leader DP (2013). FlyAtlas: database of gene expression in the tissues of Drosophila melanogaster. Nucleic Acids Res..

[56] Deddouche S (2008). The DExD/H-box helicase Dicer-2 mediates the induction of antiviral activity in drosophila. Nat. Immunol..

[57] Schneider DS, Ayres JS (2008). Two ways to survive infection: what resistance and tolerance can teach us about treating infectious diseases. Nat. Rev. Immunol..

[58] Ayres JS, Schneider DS (2012). Tolerance of infections. Annu. Rev. Immunol..

[59] Medzhitov R, Schneider DS, Soares MP (2012). Disease Tolerance as a Defense Strategy. Science.

[60] Binns D (2006). An intimate collaboration between peroxisomes and lipid bodies. J. Cell Biol..

[61] Dixit E (2010). Peroxisomes are signaling platforms for antiviral innate immunity. Cell.

[62] Weinberg SE, Sena LA, Chandel NS (2015). Mitochondria in the regulation of innate and adaptive immunity. Immunity.

[63] Odendall C (2014). Diverse intracellular pathogens activate type III interferon expression from peroxisomes. Nat. Immunol..

[64] Di Cara F, Sheshachalam A, Braverman NE, Rachubinski RA, Simmonds AJ (2017). Peroxisome-Mediated Metabolism Is Required for Immune Response to Microbial Infection. Immunity.

[65] Mast FD (2011). A Drosophila model for the Zellweger spectrum of peroxisome biogenesis disorders. Dis. Model. Mech..

[66] Nakayama M (2011). Drosophila carrying pex3 or pex16 mutations are models of Zellweger syndrome that reflect its symptoms associated with the absence of peroxisomes. Plos One.

