# Supplementary material for: Peroxisome-associated Sgroppino links fat metabolism with survival after RNA virus infection in Drosophila
Source: Sci Rep. 2019 Feb 14;9:2065. doi: 10.1038/s41598-019-38559-x (PMC6375949; doi:10.1038/s41598-019-38559-x)
Supplement: Supplementary file 1 — Supplemental Data [file 41598_2019_38559_MOESM1_ESM.pdf]

Supplementary figures and information

Peroxisome-associated *Sgroppino* links fat metabolism with survival after RNA virus infection in *Drosophila*

Sarah H. Merkling, Human Riahi, Gijs J. Overheul, Annette Schenck, and Ronald P. van Rij[1]

Supplemental data

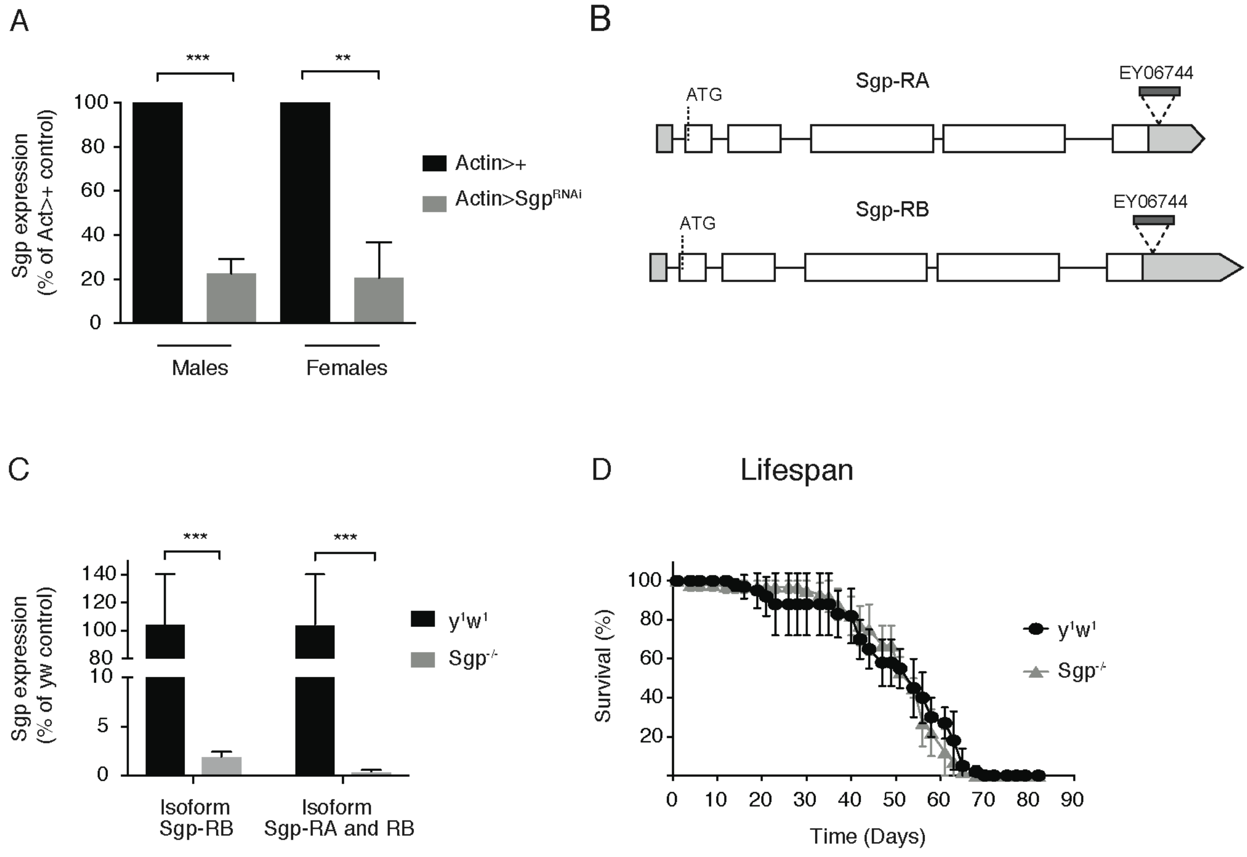

Supplementary Figure 1. *Sgp* expression levels are reduced in *Sgp*^{RNAi} and *Sgp* mutant lines, and *Sgp* deficiency does not alter life-span.

(**A**) Relative *Sgp* expression levels in flies expressing *Actin-Gal4>UAS-Sgp*^{RNAi} (Actin>Sgp^{RNAi}) compared to flies expressing *Actin-Gal4* only (Actin>+). Expression of *Sgp* was normalized to transcript levels of the housekeeping gene *Ribosomal Protein 49* and expressed as percentage of *Actin-Gal4>+*. (**B**) Structure of the *Sgroppino* locus. Both isoforms (Sgp-RA and Sgp-RB) are represented. Boxes represent exons (5' and 3'-untranslated regions in gray, and coding sequence in white). The EY06744 insertion site of a P{EPgy2} transposable element is depicted by dashed lines. (**C**) Relative *Sgp* expression levels in flies carrying the EY06744 transposon insertion in the 3'-untranslated region of the *Sgp* transcript (*Sgp*$^{-/-}$). Expression of *Sgp* was normalized to transcript levels of the housekeeping gene *Ribosomal Protein 49* and expressed relative to wild-type control flies (y^1w^1). (**D**) Lifespan of *Sgp* mutant and y^1w^1 wild-type flies at 25°C. Data represent means and s.d. of three biological replicates of (**A**, **C**) 5 or (**D**) 20 flies for each genotype. Student's t-tests were used to compare differences in *Sgp* expression (**$P < 0.01$, ***$P < 0.001$).

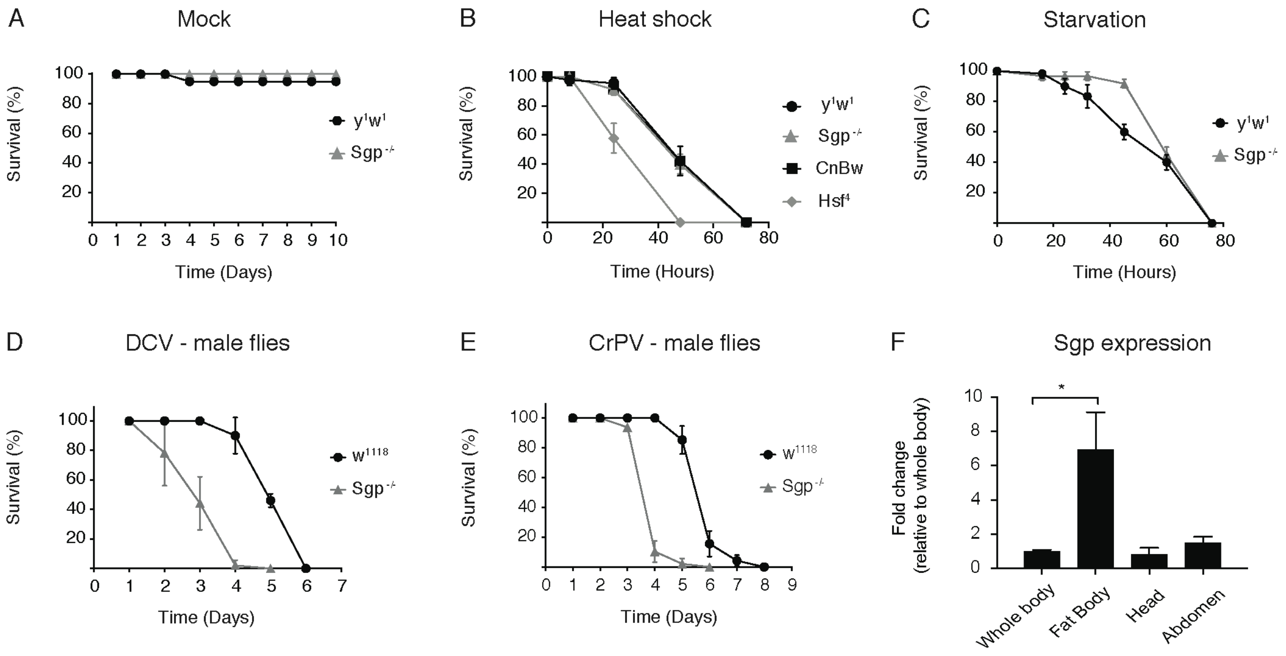

Supplementary Figure 2. *Sgp* mutant flies are not sensitive to abiotic stressors, and hypersensitivity of *Sgp* mutant flies to RNA virus infection is not sex-dependent.

(**A**) Survival of wild-type (y^1w^1) and *Sgp* mutant flies following inoculation with Tris buffer that serves as a mock control for the infections of Figure 1. (**B**) Survival upon heat shock at 35°C of *Sgp* and *Heat shock factor* (*Hsf*) mutant flies, compared to their wild-type controls, y^1w^1 and *CnBw*, respectively. (**C**) Survival of *Sgp* mutants and control flies upon starvation. (**D**, **E**) Survival of male *Sgp* mutants and w^{1118} control flies upon (**D**) DCV and (**E**) CrPV infection. (**F**) Expression levels of *Sgp* measured by RT-qPCR on whole bodies, heads, abdomens, or fat bodies of y^1w^1 flies. Expression of *Sgp* was normalized to transcript levels of the housekeeping gene *Ribosomal Protein 49* and expressed relative to whole bodies. Data represent means and s.d. of three biological replicates of (**B-F**) 15 flies for each genotype. Panel **A** are data from one pool of 20 flies per genotype. A Student's t-test was used to compare the difference in *Sgp* expression (*$P < 0.05$).

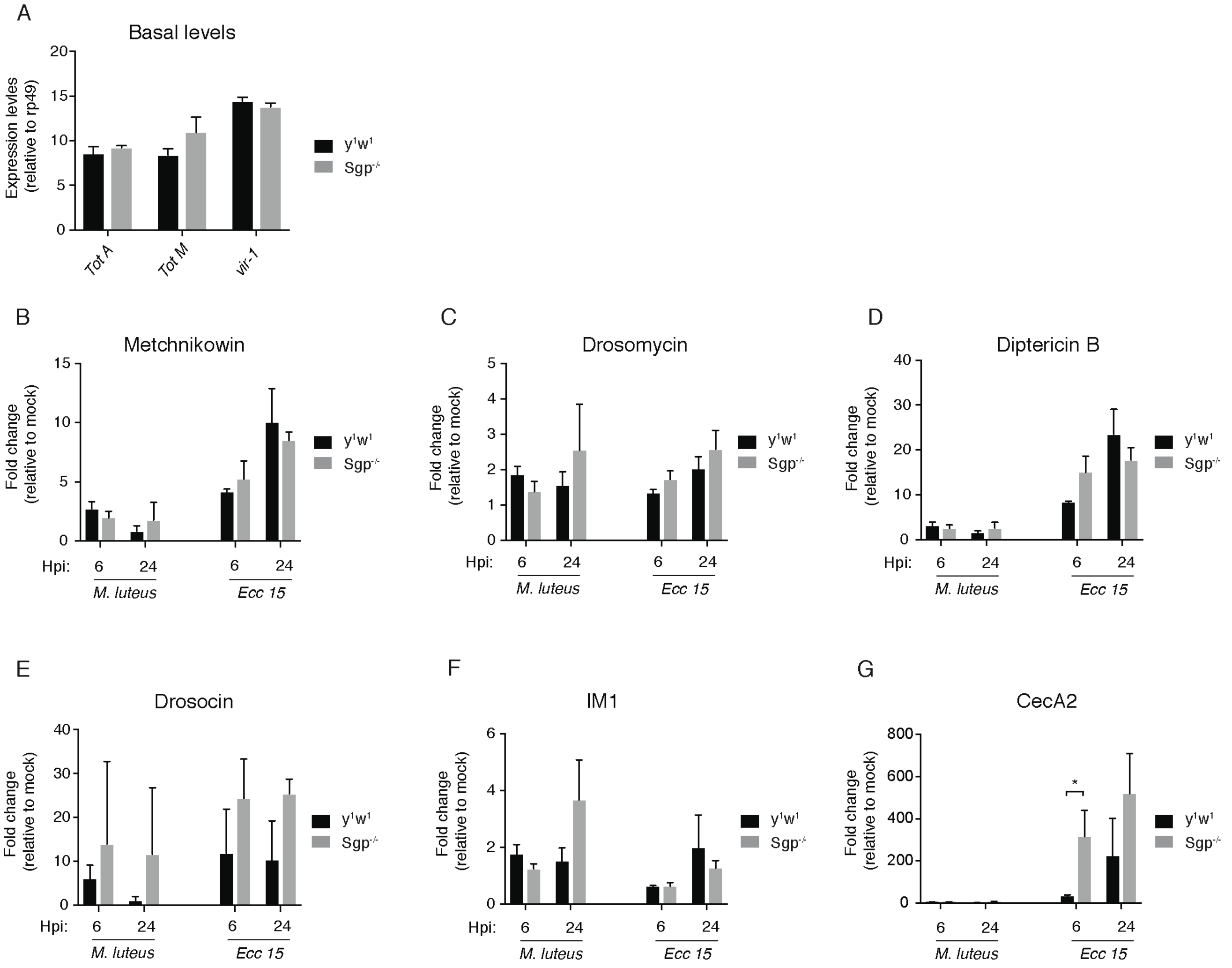

Supplementary Figure 3. (A) Constitutive expression levels of the Jak-Stat dependent transcripts *TotM*, *TotA*, and *vir-1* determined by RT-qPCR in 3 to 5 day-old wild-type or *Sgp* mutant flies. Expression of the indicated genes was normalized to transcript levels of the housekeeping gene *Ribosomal Protein 49*. **(B-G)** Expression of NF-κB dependent immune genes at 6 and 24 hours after pricking with a needle dipped in a suspension (OD_{600}=100) of *Micrococcus luteus* (Gram positive) and *Erwinia caratovora caratovora 15* (*Ecc 15*, Gram negative) determined by RT-qPCR in wild-type or *Sgp* mutant flies. Expression of the indicated genes was normalized to transcript levels of the housekeeping gene *Ribosomal Protein 49* and expressed as fold change relative to mock prick (Tris buffer). Data are means and s.d. of three independent pools of 10 female flies for each genotype. Differences in expression were not significantly different in Student's t-tests between *Sgp* mutants and control flies, at both time points and upon both challenges, with the only exception of CeCA2 expression upon Ecc 15 challenge at 6 hpi.

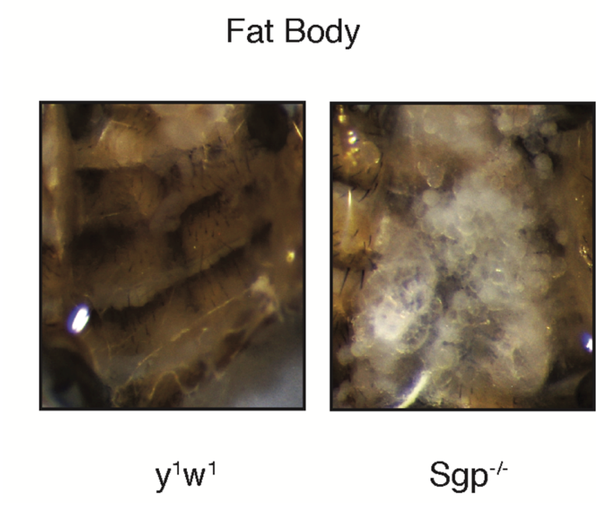

Supplementary Figure 4.
Abdominal carcasses after removal of digestive and reproductive organs of wild-type and *Sgp* mutant flies visualized using a stereomicroscope.

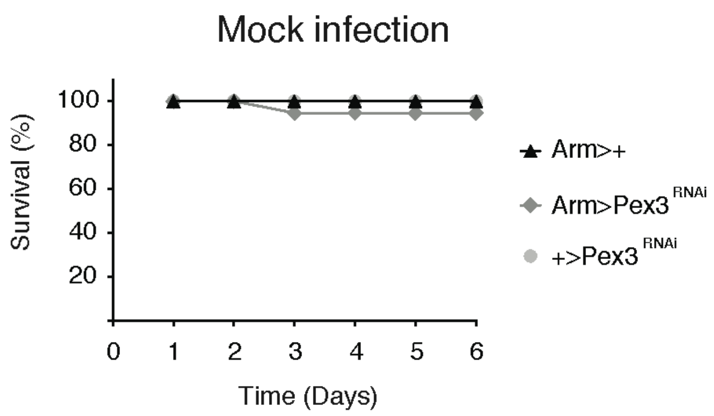

Supplementary Figure 5. Survival upon mock infection of flies expressing an RNAi-inducing hairpin RNA targeting *Pex3*. The ubiquitous *armadillo* driver (*arm-Gal4*) was used to drive expression of the transcription factor Gal4, which binds the Upstream Activating Sequence to induce expression of a short hairpin RNA targeting *Pex3* (Arm>Pex3^{RNAi}). Data represent a minimum of 15 female flies for each genotype.

Supplementary Table 1: List of significantly upregulated genes (> 2-fold) in *G9a* mutant flies infected with DCV versus mock at 24 hours post-infection. Fold change in expression in the corresponding wild-type context is shown in the last column, if significant. The full list of significantly up- and down-regulated genes in wild-type flies has previously been published [25]. The full data set is available at the NCBI Gene Expression Omnibus database (accession number GSE56013), and the analysis was previously described [21].

Symbol	*Adjusted p-value*	*Fold Change (DCV vs mock infection in G9a mutants)*	*Fold Change (DCV vs mock infection in wild-type flies)*
Hsp70Bb	1,75E-104	22,9	5,90
Hsp70Bbb	8,43E-109	22,5	5,98
Hsp70Bc	1,79E-119	22,3	6,11
Hsp70Ba	2,66E-127	22,2	5, 86
Hsp70Ab	6,23E-46	18,8	9,45
Hsp70Aa	6,23E-46	18,8	9 ,51
Hsp68	9,25E-141	17,3	4,03
CG32368	2,79E-28	11,8	n.s.
CG33926	4,95E-18	7,1	n.s.
Vago	2,65E-35	6,6	n.s.
CG13091	3,12E-14	5,9	n.s.
Spn88Eb	5,30E-27	5,9	n.s.
vir-1	8,66E-31	5,5	n.s.
Socs36E	4,85E-25	5,3	2,20
stv	2,35E-30	4,8	n.s.
Lsd-1	9,01E-32	4,1	2,68
Mmp1	1,52E-09	3,9	n.s.
lectin-37Da	2,76E-04	3,8	2,10
CG10910	7,30E-09	3,8	n.s.
CG13641	5,36E-05	3,7	n.s.
CG15043	4,46E-09	3,6	n.s.
CG13324	3,26E-04	3,6	n.s.
h	1,11E-20	3,5	n.s.
Tsp42Eb/CG30160	1,07E-10	3,4	n.s.
CG10943	3,57E-02	3,3	n.s.
CG6912	1,85E-03	3,0	n.s.
CG11501	1,86E-04	3,0	20,97
Mur29B	6,47E-12	2,9	n.s.
Obp99b	1,86E-01	2,9	n.s.
CG5550	1,36E-01	2,9	n.s
nAcRbeta-21C	3,99E-03	2,8	n.s.
CG10912	3,90E-07	2,8	n.s.
CecB	4,99E-01	2,8	n.s.
CG9989	1,00E-02	2,8	n.s
CG10911	5,41E-11	2,8	n.s.
pst	3,77E-10	2,7	n.s.
Damm	9,19E-02	2,6	n.s.
Npc2e	7,63E-01	2,6	n.s.

Cp18	5,56E-07	2,6	n.s
CG11671	3,04E-01	2,6	n.s.
Gadd45	1,54E-03	2,5	n.s.
Rel	2,47E-09	2,5	n.s.
CG15721	7,58E-04	2,5	n.s.
Cp15	1,37E-04	2,5	n.s.
CG15422	1,00E+00	2,4	n.s.
CG5724	1,65E-01	2,4	n.s.
CecC	3,04E-01	2,4	n.s.
Muc68D	7,94E-04	2,4	n.s.
TotM	2,52E-06	2,3	13,27
CG31704	2,59E-01	2,3	2,77
CG6188	1,22E-02	2,3	n.s.
v(2)k05816	5,32E-05	2,3	n.s.
CG17264	7,44E-02	2,2	n.s.
vri	1,03E-04	2,2	n.s.
Hsp67Bb/Hsp22	8,18E-06	2,2	n.s.
CG13482	2,91E-01	2,2	n.s.
CG8147	3,09E-01	2,2	n.s.
CG16978	8,66E-02	2,1	n.s.
PGRP-SA	2,25E-01	2,1	n.s.
CG13323	2,83E-03	2,1	n.s.
CG7816	1,15E-04	2,1	n.s.
snRNA:U6:96Aa	1,00E+00	2,1	n.s.
CG8534	7,68E-01	2,1	n.s.
CG5118	2,84E-03	2,1	n.s.
CG8965	1,67E-01	2,1	n.s.
peb	1,21E-03	2,1	n.s.
dnr1	1,43E-03	2,0	n.s.
CG34176	8,95E-01	2,0	n.s.
CG4269	5,68E-01	2,0	n.s.
sug	7,03E-04	2,0	n.s.
Hsp23	1,61E-01	2,0	n.s.
CG15673	4,86E-01	2,0	n.s.
Msp-300	3,25E-04	2,0	n.s.
Fst	6,70E-01	2,0	3,34 (down-regulated)

Supplementary table 2: List of primers

Application	Primer name	Sequence (5'-3')
RT-qPCR	Rp49 fwd	ATGACCATCCGCCCAGCATAC
	Rp49 rev	CTGCATGAGCAGGACCTCCA
	DCV fwd	TTGCCATTGCACCACTAAAA
	DCV rev	AAAATTTCGTTTTAGCCCAGAA
	CrPV fwd	AGCTTGGATCTCAGCGAAAG
	CrPV rev	GAGCCCGCTGAGATGTAAAG
	FHV fwd	ACCTCGATGGCAGGGTTT
	FHV rev	CAAAAGGCCATGGTTCAAG
	DXV fwd	CATCGTCGACATCACCAAAC
	DXV rev	ACAACGGATCCTGTGAAAGC
	IIV-6 (193R) fwd	CACAACCAAGATTTGGATCACAACCA
	IIV-6 (193R) rev	ACACGAAGAATGACCACAAGGA
	Vago fwd	CAGCCAAGCGATTCCTTATC
	Vago rev	CTCATACAGTGGGCAGCATC
	vir-1 fwd	ATTACTCCGAATTCGAAGCTTCC
	vir-1 rev	CGAATTCTTCACGCTCCTTC
	Listericin fwd	TTGCGGCCATTCTGGCCATG
	Listericin rev	TTTACGTCCCCAACTGGAAC
	TotA fwd	CCCTGAGGAACGGGAGAGTA
	TotA rev	CTTTCCAACGATCCTCGCCT
	TotM fwd	ACCGGAACATCGACAGCC
	TotM rev	CCAGAATCCGCCTTGTGC
	Drosomycin fwd	GTACTTGTTCGCCCTCTTCG
	Drosomycin rev	ACAGGTCTCGTTGTCCCAGA
	Metchnikowin fwd	TACATCAGTGCTGGCAGAGC
	Metchnikowin rev	AATAAATTGGACCCGGTCTTG
	Diptericin B fwd	TGTGAATCTGCAGCCTGAAC
	Diptericin B rev	GCTCAGATCGAATCCTTGCT
	Drosocin fwd	CAAGCCACGCCCCTACAG
	Drosocin rev	GGCAGCTTGAGTCAGGTGAT
	Immune induced 1 fwd	GCCCAGTGCACTCAGTATCC
	Immune induced 1 rev	TCGAATCCTTGGGTTGAAAC
	Cecropin A2 fwd	CATCAGAGCTATAGCTACTC
	Cecropin A2 rev	GTCCCTGGATTGTGGCGTCG
	Pex3 fwd	ACTGGCAACCTATGTCTACGC
	Pex3 rev	AGGGAGAGATACTGCTGCTTTAG
	Sgp fwd	TGGCATCCGATTGTTAGGGG
	Sgp Rev (RB)	CAATCGAATTACGCACGAGC
	Sgp Rev (RA and RB)	ACGCACGAGCATATAACTTT
Cloning	Tag-EGFP-KpnI*	AGTggtaccCAACATGGTGAGCAAGGGCGAG
	Tag-EGFP-EcoRI*	GGTgaattcCTTGTACAGCTCGTCCATGC
	Tag-RFP-Acc65I*	ACTGcgtacgAACATGGTGTCTAAGGGCGAAGAG
	Tag-RFP-NotI*	CAGTgcggccgcTTATCTAGATCCGGTGGATC
	Sgp-SacI*	ACTGgagctcAAATGCAGACAGACATATTGC
	Sgp-SacI*	TGACgagctcTTAAATACTTGGAAAATCGGCC
	PMP34-XbaI*	ACTGtctagaATGGTGGCCCCCTCGAAAC
	PMP34-SacI*	TGACgagctcTCAGTTGCGCTTAAGCAGC
dsRNA synthesis	T7-PMP34 fwd	TAATACGACTCACTATAGGGAGACGGCGATGTGCGGAGTACAAG
	T7-PMP34 rev	TAATACGACTCACTATAGGGAGACCCAAAAGGGTGTGGTGGTG
	T7-dsLuc fwd	TAATACGACTCACTATAGGGAGATATGAAGAGATACGCCCTGGTT
	T7-dsLuc rev	TAATACGACTCACTATAGGGAGATAAAACCGGGAGGTAGATGAGA
	T7-dsSgp fwd	TAATACGACTCACTATAGGGAGAATTTGGTATCCATTTGCCCAC
	T7-dsSgp rev	TAATACGACTCACTATAGGGAGATCCTTGTAAAGGAACACCCG
	T7-dsPex3 fwd	TAATACGACTCACTATAGGGAGAACCATCTTTGCGGCACGTTATG
	T7-dsPex3 rev	TAATACGACTCACTATAGGGAGAAGCGGTATGGTCTTGGTGAC

* Restriction sites are written in lowercase
